# Two Invasive Thymomas Incidentally Found during Coronary Artery Bypass Graft Surgery

**DOI:** 10.1155/2016/1516521

**Published:** 2016-10-31

**Authors:** Navid Omidifar, Maral Mokhtari, Mansoureh Shokripour

**Affiliations:** ^1^Department of Pathology, School of Medicine, Yasuj University of Medical Sciences, Yasuj, Iran; ^2^Quality Improvement in Clinical Education Research Center, Shiraz University of Medical Sciences, Shiraz, Iran; ^3^Department of Pathology, School of Medicine, Shiraz University of Medical Sciences, Faghihee Hospital, Shiraz, Iran

## Abstract

Thymoma, the most common neoplasm of the anterior mediastinum, is a rare tumor of thymic epithelium that can be locally invasive. We reported 2 cases of invasive thymoma incidentally found during routine coronary artery bypass graft (CABG) surgery at Faghihee Hospital of Shiraz University of Medical Sciences of Iran in a period of about 6 months. The 2 patients were male and above 60 years old. They had no clinical symptoms and radiological evidence of mediastinal mass before detection of the tumor during operation. For both patients mass was completely excised and sent to the laboratory. The ultimate pathological diagnosis of both masses was invasive thymoma (stage 2). There are few reports in which thymomas were found incidentally during cardiac surgery. In spite of rare coincidence, due to being asymptomatic and possibly invasive, special attention to thymus gland during cardiac surgery or other mediastinal surgery and preoperative imaging studies seem to be reasonable approach.

## 1. Introduction

Thymomas are rare tumors arising from thymic epithelium, characterized by neoplastic epithelial proliferation in the background of nonneoplastic lymphocytes called thymocytes. Mostly, they present with anterior mediastinal mass [1]. The overall incidence of thymoma is about 0.15 per 100000 [2]. The most common symptoms are chest pain, cough, and dyspnea due to pressure effect of mediastinal tumor [2, 3]. They may also present with symptoms related to intrathoracic spread or paraneoplastic disorders such as myasthenia gravis, red cell aplasia, and pemphigus [3]. But nowadays because of increased utilization of computed tomography (CT) for other diseases, detection and diagnosis of asymptomatic patients with incidentally identified thymoma have been increased [4]. Chest radiography is the initial method for approach of patients suspected to have thymoma; by this simple imaging about 45 to 80 percent of these patients are detectable [4]. However, for more definite diagnosis and for staging before operation and before final pathological diagnosis, CT scan should be performed [4, 5].

Thymoma is a bland looking tumor and does not have cytologic features of carcinoma but can be locally invasive with recurrence [1]. This tumor often has a thick fibrous capsule that makes a lobular appearance for the tumor [5]. About 30 to 40% of thymomas show invasion macroscopically into surrounding soft tissue or microscopically through tumor capsule [5]. These thymomas were the so-called malignant thymoma traditionally. The most effective treatment of thymoma is complete surgical resection of the tumor that also defines the prognosis of the patients. Adjuvant radiation therapy is controversial but some studies showed lower rates of recurrence [5].

There are few reports in which thymic mass was discovered incidentally during cardiovascular workups prior to coronary artery bypass grafting (CABG)/stenting or intraoperatively [6–10].

Here in our report 2 cases of invasive thymomas were found incidentally during urgent CABG surgery that demonstrated, in spite of rare coincidence, that special attention to thymus gland during cardiac surgery or other mediastinal surgery or preoperative imaging studies should be given. These asymptomatic tumors discovered during surgery need appropriate management.

## 2. Patients' Description

### 2.1. Patient 1

**Figure 1:**
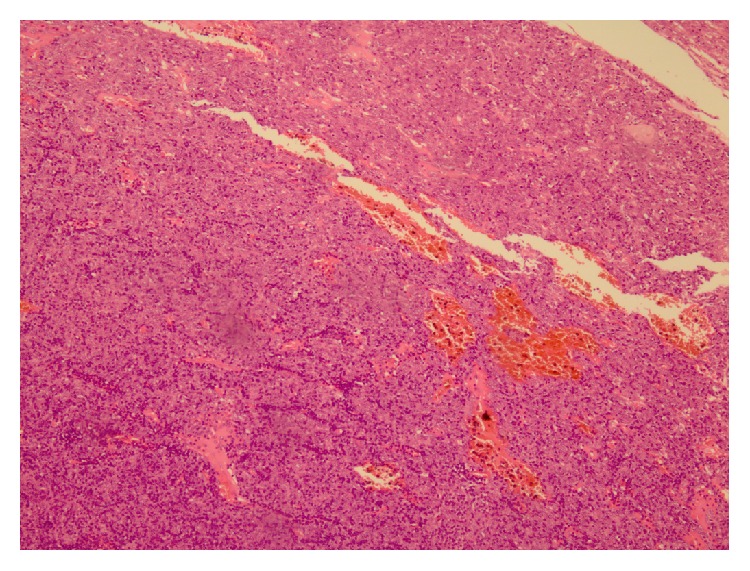
Microscopic view of first patient's sample. It shows prominent epithelial component that is consistent with B_3_ type WHO classification 2004 (H&E stain ×200).

**Figure 2:**
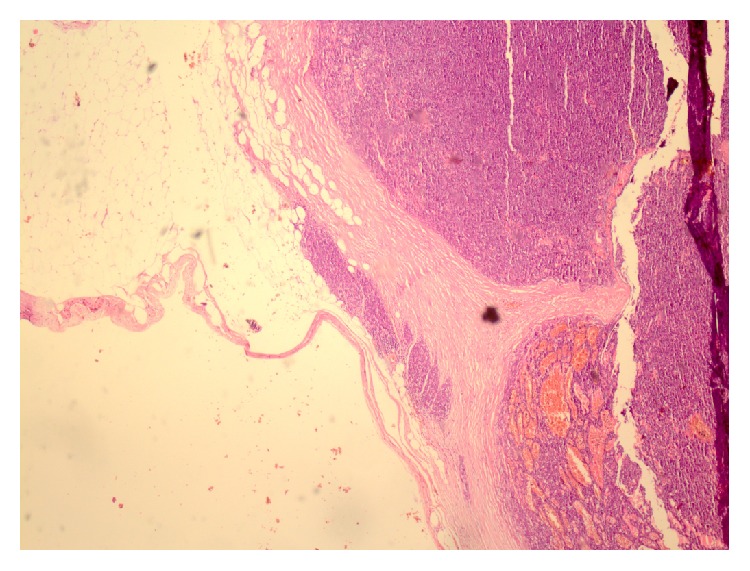
Microscopic view of first patient's sample. Peritumoral fat shows a small microscopic focus of invasion (H&E stain ×40).

The patient was 63-year-old male with coronary artery disease who underwent CABG surgery without any other past medical history like muscle weakness or pressure effect of mediastinal mass. Physical and routine laboratory tests were normal. The patient was a case of three-vessel disease. An anterior mediastinal mass with largest dimension of 1.5 centimeter attached to pericardium was found during surgery. The mass was partially encapsulated. All of the tissue was embedded for pathologic examination. Routine chest X ray before surgery showed no significant abnormality. Our histopathological diagnosis of the patient that was confirmed by immunohistochemistry study (IHC) was thymoma with microscopic invasion (stage 2) and predominant epithelial component (proved by positivity for 34beta E12 cytokeratin). The absence of staining for CD34 and vimentin in correlation with histomorphologic evaluation ruled out epithelioid sarcoma. Background T cells were positive for CD3 and CD5. The type of tumor determined B_3_, WHO classification 2004 (Figures 1 and 2).

After complete surgical excision he was recommended to get chemotherapy and radiotherapy as the treatment for the patient. After 2 years of follow-up he had no sign or symptom of recurrence.

### 2.2. Patient 2

**Figure 3:**
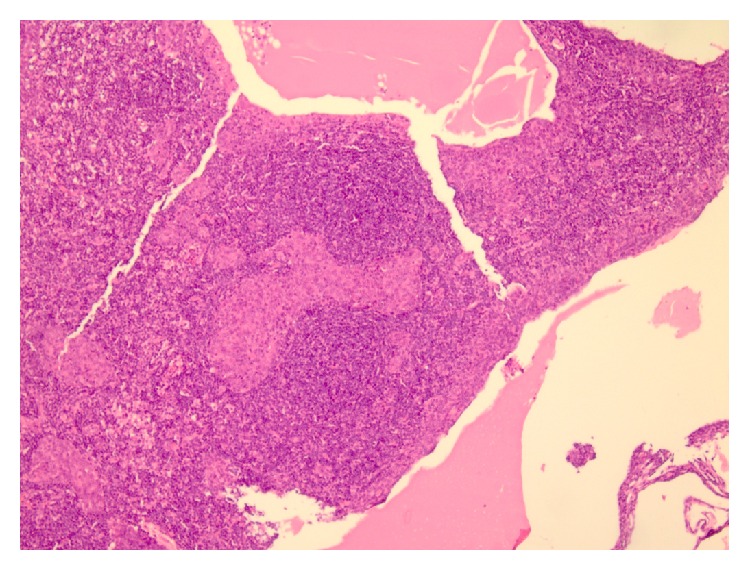
Microscopic view of second patient's sample. Discrete epithelial nodules with B lymphocytes in follicular figures with microcystic changes are evident; compatible with Micronodular Thymoma WHO classification 2004 (H&E stain ×200).

**Figure 4:**
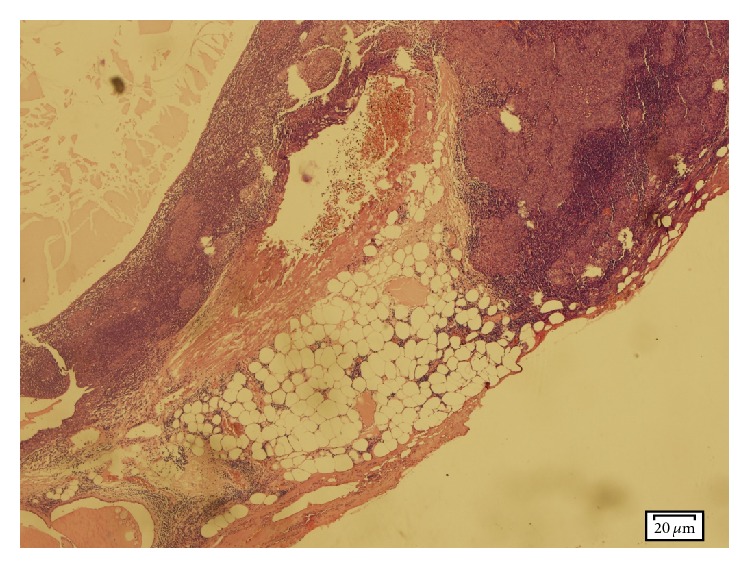
Microscopic view of second patient's sample. It shows microscopic focus of invasion, microcystic formation, and discrete and confluent epithelial nodules (H&E stain ×200).

The other patient, a 67-year-old male with history of coronary artery disease and MI of many years ago, was admitted for open heart surgery. Like the first patient, this patient had no history of pressure effect of anterior mediastinal mass or muscle weakness. There was no radiologic finding of mediastinal mass in chest X ray before surgery. CT scan has not been performed before surgery. During open heart surgery a well-defined mass with 1.5 centimeter in largest dimension was identified in front of heart in anterior mediastinum with impression of lymph node. Mass was completely excised and sent to our pathology laboratory. In cut sections the mass had cystic and solid appearance (totally embedded). The pathologic diagnosis of this patient was invasive thymoma with the same stage as previous patient's tumor (stage 2). Microscopically it consisted of multiple, distinct, or confluent epithelial nodules separated by an abundant B lymphocytes that were shaped in follicles. The epithelial nodules were composed of mostly plump spindle cells with bland looking oval nuclei. Nodules comprised few scattered lymphocytes. No Hassall corpuscles were seen. Mitotic figure was absent or just a few ones. Areas of micro- and macrocysts were identified. IHC study showed positivity for 34beta E12 cytokeratin in epithelial component. CD20 was positive in majority of the lymphocytes, but mature CD5+ T cells were also seen. Few intraepithelial lymphocytes were identified. B-cells formed follicles without germinal centers. The diagnosis was made as Micronodular Thymoma, WHO classification 2004 (Figures 3 and 4).

The patient did not receive any other treatment such as chemotherapy or radiotherapy after surgery and had no complaint after 2 years of follow-up.

## 3. Discussion

Thymomas are rare epithelial tumors of thymus gland and they represent the most common anterior mediastinal mass [3]. Since the thymoma may be asymptomatic or accompanied by nonspecific signs, it is not uncommon to find thymic tumors coincidentally on imaging studies for investigating other disorders [4]; moreover, some thymic tumors may be discovered intraoperatively and are not detected in radiography before surgery especially when we do not keep it as a differential diagnosis in our minds [6–10]. There are few reports in which thymomas were found incidentally during cardiac surgery and had not been detected in imaging prior to surgery [9, 10].

Histologically, thymomas are categorized in 5 types according to WHO classification; A, AB, B_1_, B_2_, and B_3_ based on the morphology of epithelial component and proportion of lymphocytes and epithelial cells [3]. These types of thymoma had no cytologic evidence of malignancy that are seen in thymic carcinoma (WHO classification type C) but they could have malignant behavior as invasiveness [5]. These different subtypes of thymoma have different potential for local invasion and recurrence and metastasis, as follows: A < AB < B_1_ < B_2_ < B_3_ < C.

Micronodular Thymoma with lymphoid stroma (MNT) is a rare entity accounting for only about 1–5% of all thymomas. The age at diagnosis ranged between 45 and 95 years. MNT is encapsulated (>90%) or minimally invasive. Grossly it is mostly in cystic shape. Microscopically, it consists of epithelial nodules that are separated by B lymphocytes. These lymphocytes sometimes shape follicles with conspicuous germinal centers. The epithelial cells are mostly spindle or oval-shaped cells with bland nuclei that resemble type A thymoma [11].

According to Masaoka staging system, four stages have been defined for thymoma. Stage 1 is a completely encapsulated tumor with no microscopic or macroscopic evidence of invasion. In stage 2 there is microscopic capsular invasion or macroscopic perithymic fat or pleural invasion. Stage 3 shows macroscopic invasion to near organs such as pericardium, great vessels, or lung. The tumors are in stage 4 when disseminated via pleura or pericardium or via hematogenous or lymphogenous roots [5].

In our report, the two tumors had no cytologic evidence of malignancy but showed small foci of microscopic invasion beyond tumor capsule into perithymic fat so they have been categorized in stage 2a (microscopical invasion). The effectiveness of mediastinal radiotherapy after surgery in stage 2 patients has not been completely proved. Our patients received no additional treatment after thymectomy and had no complaint after 2 years of follow-up.

Like our study, the Al-Smady case report study in Saudi reported two incidental invasive thymomas that are treated by thymectomy. No recurrence was observed after 2 years of follow-up. Time gap of emergence between two cases was longer than what we had (one year versus 6 months in our study).

## 4. Conclusion

We reported 2 cases of thymomas, which were detected intraoperatively during urgent CABG, and all of them were treated by total thymectomy. The incidence of thymoma during CABG is ranging from 0.2% to 0.7% [9]. In spite of rare coincidence, special attention to thymus gland during cardiac surgery or preoperative imaging studies is reasonable and leads to proper treatment of thymic mass simultaneously.

## Supplementary Material

Thymomas are rare tumors. About 30 to 40% of thymomas show invasion macroscopically into surrounding soft tissue or microscopically through tumor capsule. Here in our report 2 cases of invasive thymomas were found incidentally during urgent CABG surgery. It demonstrated the need to special attention to thymus gland during cardiac surgery or other mediastinal surgery or preoperative imaging studies.
