# Supplementary material for: Two Invasive Thymomas Incidentally Found during Coronary Artery Bypass Graft Surgery
Source: Case Rep Pathol. 2016 Oct 31;2016:1516521. doi: 10.1155/2016/1516521 (PMC5107858; doi:10.1155/2016/1516521)
Supplement: Supplementary file 1 — Thymomas are rare tumors. About 30 to 40% of thymomas show invasion macroscopically into surrounding soft tissue or microscopically through tumor capsule. Here in our report 2 cases of invasive thymomas were found incidentally during urgent CABG surgery. It demonstrated the need to special attention to thymus gland during cardiac surgery or other mediastinal surgery or preoperative imaging studies. [file 1516521.f1.docx]

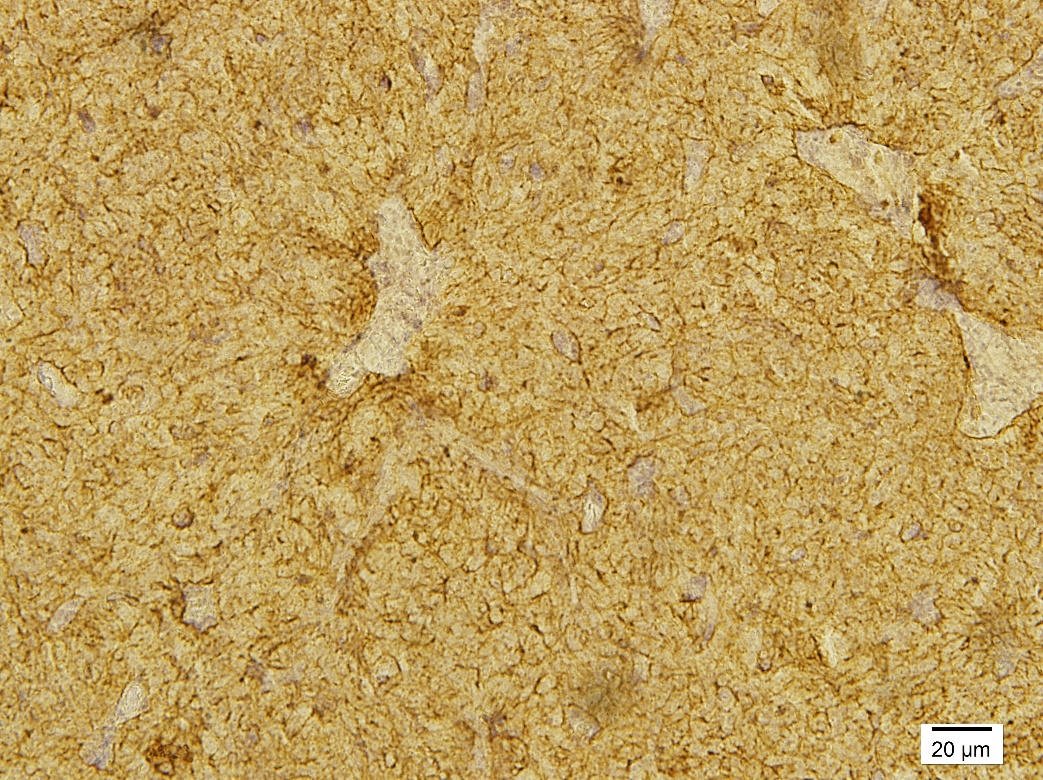


Patient 1,thymoma B3 type, cytokeratin positive


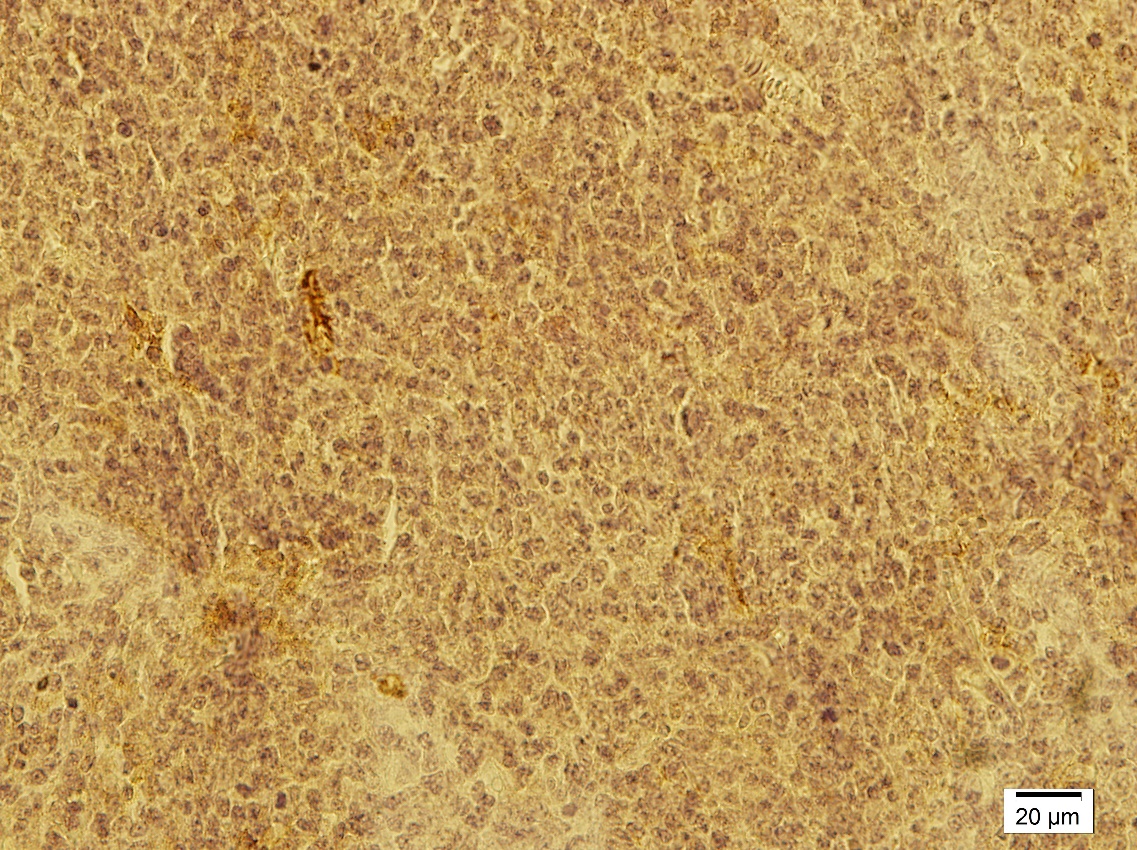


Patient 1,thymoma B3 type, CD20 negative


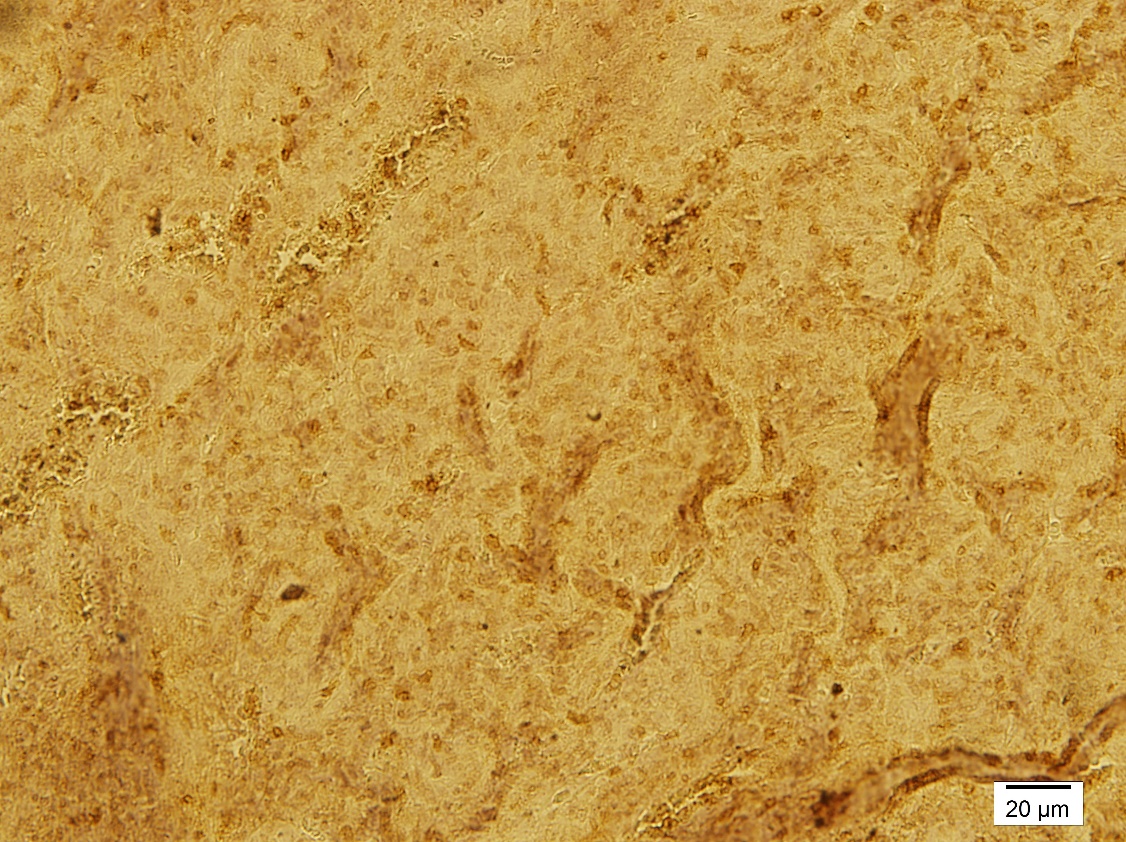


Patient 1,thymoma B3 type, CD5 positive scatterdly


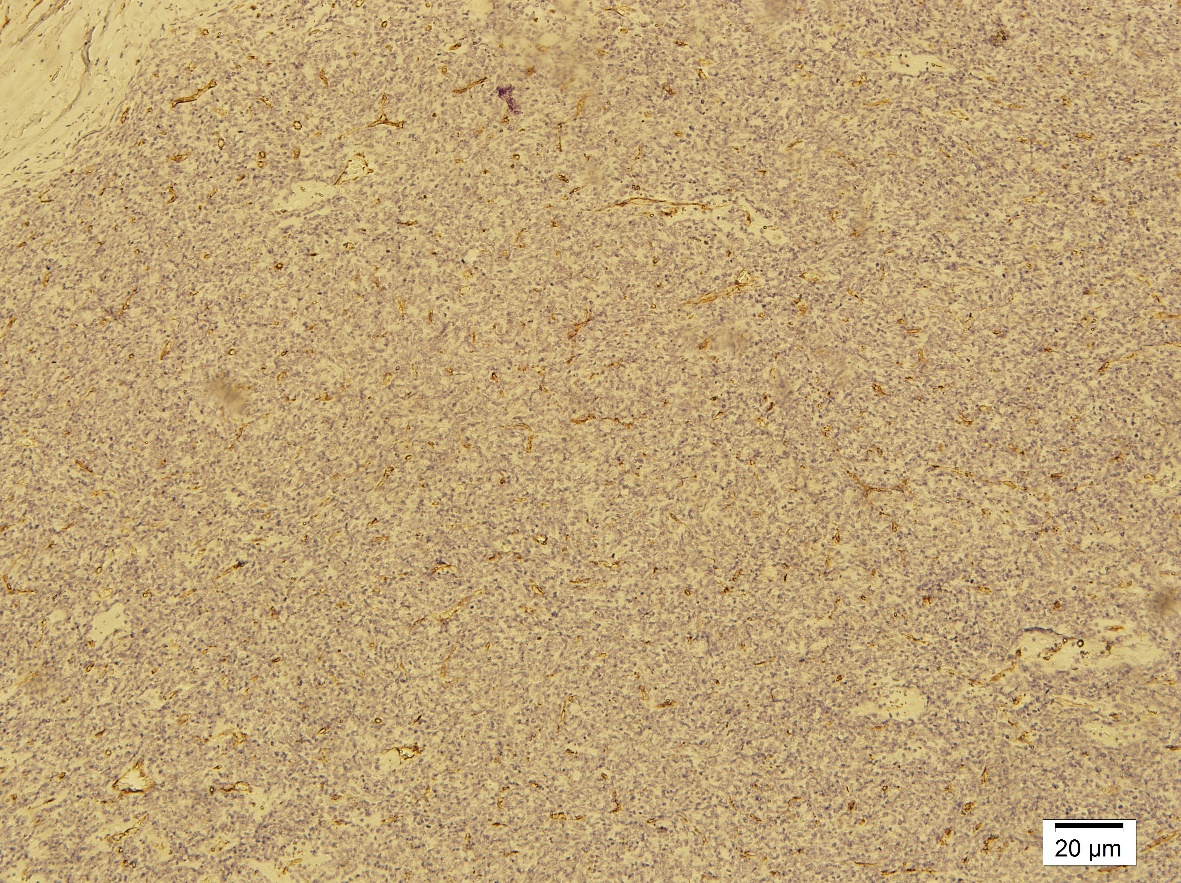


Patient 1,thymoma B3 type, CD34 negative


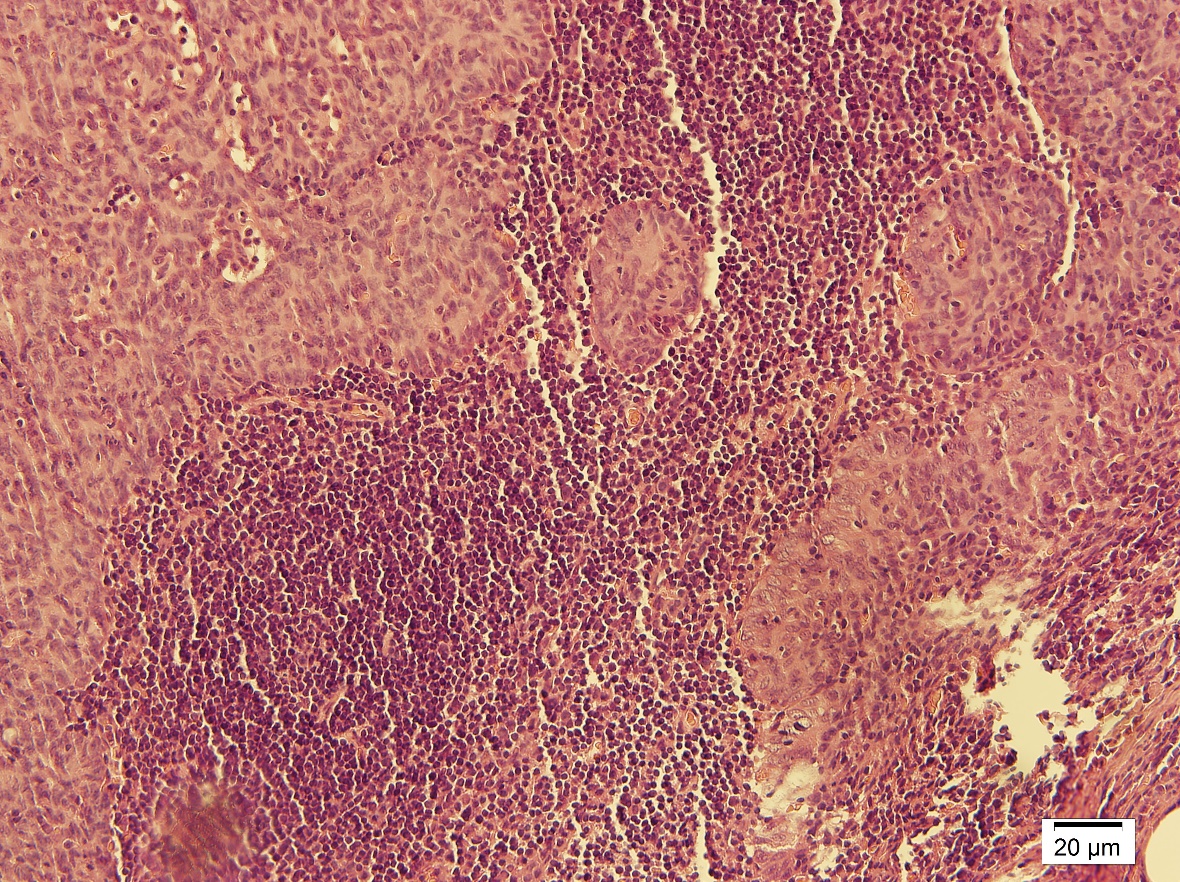


Patient 2, micronodular thymoma


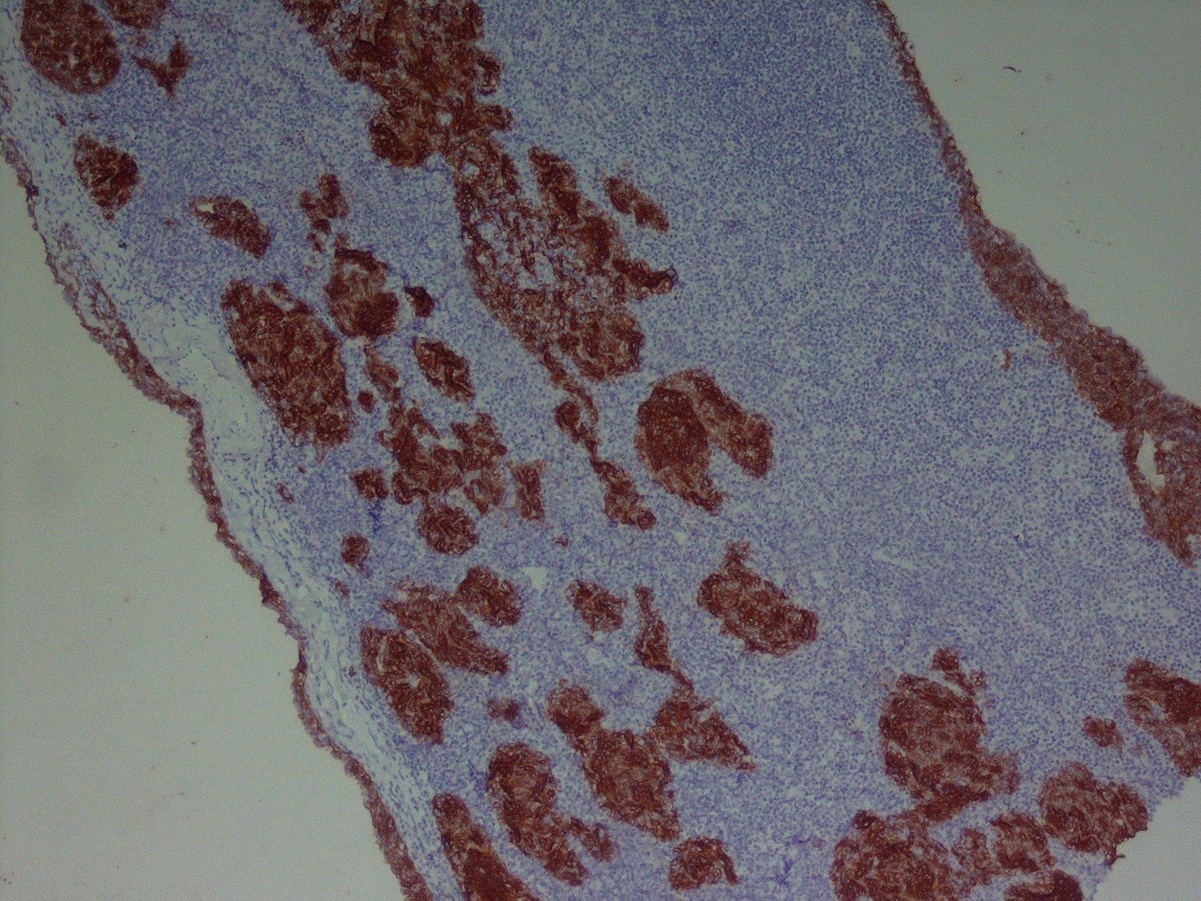


Patient 2, micronodular thymoma, CK positive in epithelial nodules


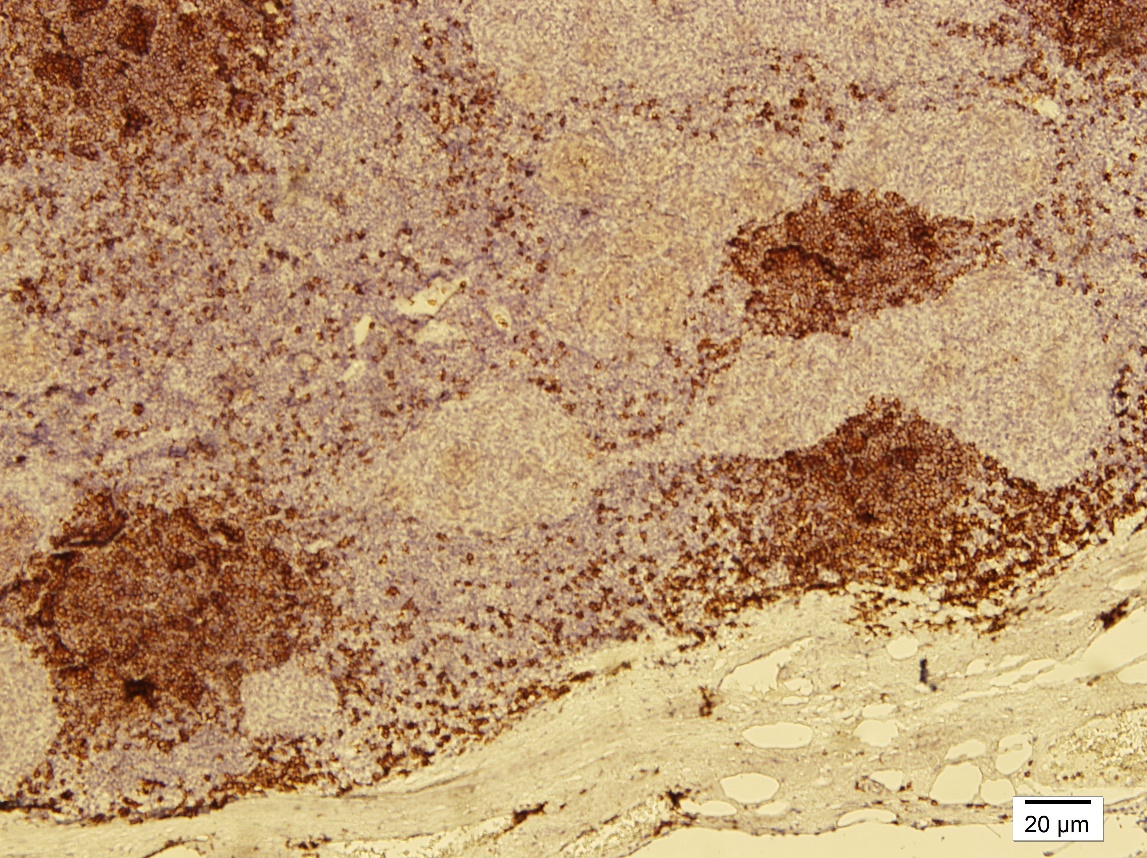


Patient 2, micronodular thymoma, CD20 positive in lymphoid follicles


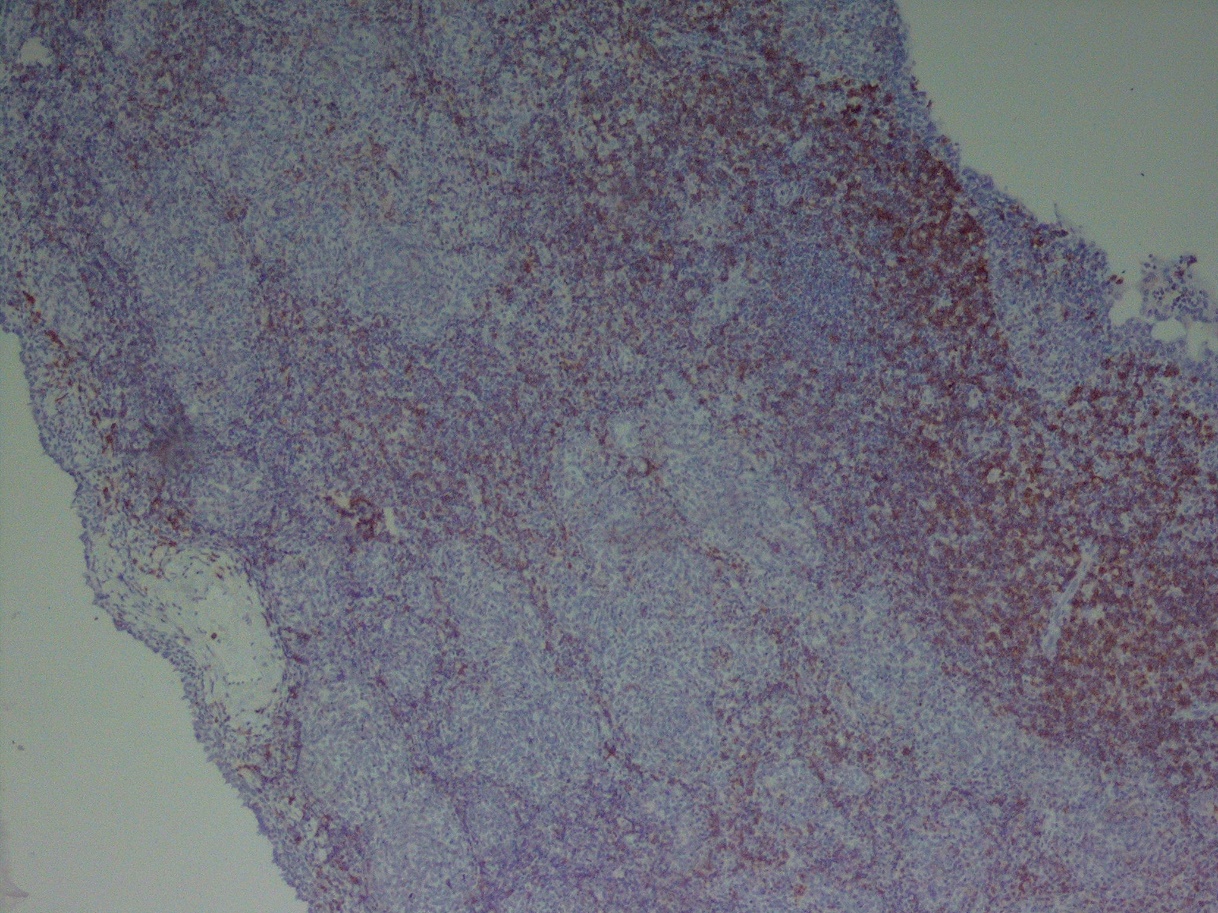


Patient 2, micronodular thymoma, CD5 positive scatterdly
